# Reactivity of Metakaolin in Alkaline Environment: Correlation of Results from Dissolution Experiments with XRD Quantifications

**DOI:** 10.3390/ma13102214

**Published:** 2020-05-12

**Authors:** Sebastian Scherb, Mathias Köberl, Nancy Beuntner, Karl-Christian Thienel, Jürgen Neubauer

**Affiliations:** 1 Civil Engineering and Environmental Science, Universität der Bundeswehr München, Werner-Heisenberg-Weg 39, 85579 Neubiberg, Germany; mathias.koeberl@unibw.de (M.K.); nancy.beuntner@unibw.de (N.B.); christian.thienel@unibw.de (K.-C.T.); 2 GeoZentrum Nordbayern, Mineralogy, Friedrich-Alexander Universitaet Erlangen-Nuernberg, Schlossgarten 5a, 91054 Erlangen, Germany; juergen.neubauer@fau.de

**Keywords:** metakaolin, degree of reaction, PONKCS, calcined clay, supplementary cementitious material, alkaline solution

## Abstract

Systematic investigation of filtrates and filter residues resulting from a 24 h treatment of metakaolin in different alkaline solutions were performed. On filtered metakaolin particles, inductively coupled plasma-optical emission spectrometry (ICP-OES) measurements reveal an enrichment of iron and titanium, which suggests an inhomogeneous distribution of these cations. Since the SiO_2_/Al_2_O_3_ ratio remains constant in all filter residues examined, the dissolution of the Si and Al monomers is congruent. Structural differences, identified by attenuated total reflection–Fourier transform infrared spectroscopy (ATR-FTIR) as a consequence of alkali uptake, influence the X-ray scattering contribution of metakaolin, and thus quantifications with the partial or no known crystal structure (PONKCS) method. This leads to deviations between the degree of reaction calculated from Si and Al solubility from filtrate and that quantified by quantitative powder X-ray diffraction (QPXRD) using the filter residue. Nevertheless, the described changes do not cause a shift in the X-ray amorphous hump in case of congruent dissolution, and thus allow the quantification of the metakaolin before and after dissolution with the same hkl-phase model.

## 1. Introduction

In recent years, the pozzolanic reactivity of calcined clays has increasingly moved into the focus of research. Their use as a supplementary cementitious material (SCM) could be a component to achieve the goal of more ecological concretes, since the replacement of cement by SCM offers one of the greatest opportunities to reduce CO_2_ emission in the production of concrete. The assessment of pozzolanic reactivity plays a crucial role in determining the possible degree of replacing cement with SCM and is directly linked to concrete strength and durability properties [1,2,3,4,5,6]. Different SCMs intervene variably in cement hydration by means of physical and chemical parameters and mechanisms [7,8]. Various scientists were investigating the question of a test method suitable for assessing the pozzolanic reactivity of different SCM directly from the respective SCM [9,10,11,12,13,14,15,16,17,18]. Another approach is the determination of the reaction degree of SCM in cementitious systems [19,20,21,22,23,24,25,26,27,28,29,30]. Different parameters such as the reactive silica content, the CaO or Ca(OH)_2_ consumption, the relative strength index or the content of soluble silicon (Si) and aluminum (Al) ions in alkaline solution are common to assess the pozzolanic reactivity. Different wet chemical, analytical and empirical methods such as thermogravimetric analysis, isothermal calorimetry, quantitative X-ray diffraction (QXRD) or compressive strength are used for the different approaches.

For calcined clays, which, in addition to silicon, can provide a considerable amount of reactive aluminum, comparison of the test methods revealed a considerable variation in the suitability of individual methods, even if they work very well in some other cases, e.g., for fly ash or slag [13,23,31,32,33,34].

The quantification of X-ray amorphous SCM with the partial or no known crystal structure (PONKCS) method [35] is a powerful tool to determine the SCM content in blended cements [36,37]. Quantification of SCM in hydrating systems is a challenging task due to strong overlap of the broad SCM peaks with X-ray amorphous hydrates and the free, not chemically bound, water [36,38]. The latest research confirms the possibility of quantifying the reacted SCM during in situ XRD measurements of hydrating systems [28]. Others show that the calculation of the degree of reaction can lead to major deviations resulting from error propagation. This depends largely on the substitution rate of the SCM and the degree of reaction [23,26,36].

Due to the high temperatures (1100–1700 °C) and fast cooling involved in the formation of fly ash, a large proportion of the particles are vitreous. The calcination of clays takes place at significantly lower temperatures (600–900 °C). Even if crystalline clay minerals are transformed into X-ray amorphous “metaphases”, their habitus and layer structure remain intact (pseudomorphosis). According to Brindley and Nakahira [39], a pseudohexagonal skeleton of [SiO_4_]-tetrahedra in the metakaolin is preserved at calcination temperatures of 600 °C. Massiot et al. [40] describe effects observed with nuclear magnetic resonance (NMR) between 450 and 850 °C as a newly formed silicon network without structural long-range order. In contrast to fly ash, in which the silicon release takes place via a solution process of the vitreous particles in alkaline environment [41], the mechanism of ion release of calcined clays is not finally investigated. Granizo et al. [42] describe the leaching kinetics of metakaolin in 5 and 8 M NaOH solution as a three-stage process. According to their hypothesis, the dissolution process is initially incongruent for a short time, followed by a longer period of congruent dissolution behavior and, finally, incongruent again. Garg and Skibsted [43] show that crystallographic defects accelerate dissolution in alkaline solution during the first hours, followed by a period where the rate of reaction is constant. They conclude that the dissolution process of metakaolin calcined at 500 °C is congruent and becomes increasingly incongruent with increasing calcination temperature. An incongruent dissolution process of metakaolin could lead to changes in the contribution of metakaolin to the diffractogram and thus falsify the quantifications with the PONKCS method. This is the starting point of this study, which intends to provide an insight into the following questions: Is there a correlation between the amount of Si- and Al-ions in alkaline solution derived from dissolution tests and the quantification of the metakaolin with the PONKCS method? How does the dissolution process of metakaolin affect its X-ray amorphous hump in the diffractogram? Although the reactivity of metakaolin has been described in numerous publications [14,15,17,27] and its content has already been quantified [26,28,36], the previous questions raised have not yet been considered. Therefore, the method of Kaps and Buchwald [11,12] is used to determine the pozzolanic reactivity of metakaolin on the basis of the solubility of Si- and Al-monomers in alkaline solution. A systematic investigation of the filter residue should provide information about the processes taking place during ion release, the impact on the metakaolin and correlations between the ions dissolved and the quantity evaluated with the PONKCS method for the first time. The contribution of this work shows the possibilities and limitations of the established methods. A better understanding contributes directly to improving the quantification with the PONKCS method and the assessment of the results obtained. Thus, the present study contributes to the evaluation of the suitability of calcined clays as SCM.

## 2. Materials and Methods

### 2.1. Materials and Test Program

The investigation deals with the solubility behavior of metakaolin (MK) in deionized water and various alkaline solutions. The flash calcined metakaolin is commercially available and was ready for use. The chemical composition was determined by inductively coupled plasma-optical emission spectrometry (ICP-OES) and the mineralogical composition by quantitative powder X-ray diffraction (QPXRD) with 20 wt.% ZnO as an internal standard to determine the X-ray amorphous content (Table 1). The data have already been published by the authors in [38,44].

**Figure 1:**
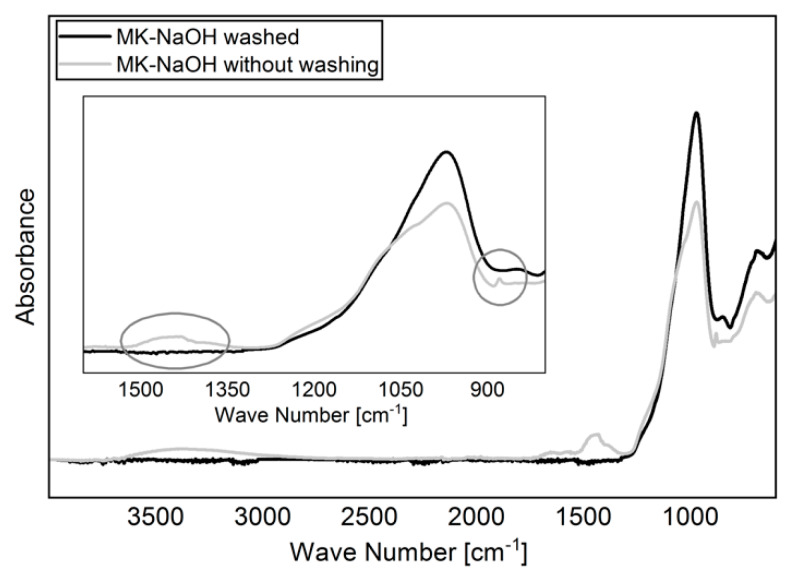
FTIR spectra of dried MK-NaOH after and without prior washing the sample. The carbonization of adhered NaOH to the particle surfaces can be avoided by washing the sample.

For the experiments, 5 g MK was shaken in 400 mL solution for 24 h on a vibrating unit. The solutions used were deionized water (H_2_O; pH = 5.8) as reference, 10 wt.% sodium hydroxide solution (NaOH; pH = 13,2), 10 wt.% potassium hydroxide solution (KOH; pH = 14.1) and a model pore solution of 100 mmol/L NaOH and 500 mmol/L KOH (MOH; pH = 13.5). The pH of the suspensions was measured after 5 min, 30 min, 6 h, and 24 h. The measurements of the pH were carried out with the digital pH meter WTWmulti 3430 with SenTix 940-3 pH-electrode (WTW, Weilheim, Germany). Two independent preparations were applied for each solution. Subsequently, the samples were filtered through a suction filter with depression. Both the filtrate and the filter residue were used for further analysis. The filter residue was first kept in the filter and washed with distilled water until the measured pH value of the water leaking from the filter was less than 8. This procedure was necessary to prevent the hydroxide solutions from adhering to the particle surfaces and falsifying further analyses of the filter residue. Finally, the filter residue was dried over night at 60 °C in a warming cabinet and carefully crushed in an agate hand mortar the next day. The differences between drying a sample without prior washing and drying a washed sample are shown in Figure 1. Analogous to other studies [45,46], the carbonization of NaOH can be clearly identified.

In addition to weighing the filter residue, following methods were used to analyze the sample:

1. Quantitative Powder X-Ray Diffraction (QPXRD);
2. Scanning Electron Microscopy/Energy Dispersive X-ray analysis (SEM/EDX);
3. Element analysis by Inductively Coupled Plasma Optical Emission Spectrometry (ICP-OES);
4. Attenuated Total Reflection–Fourier Transform Infrared spectroscopy (ATR-FTIR).

**Figure 2:**
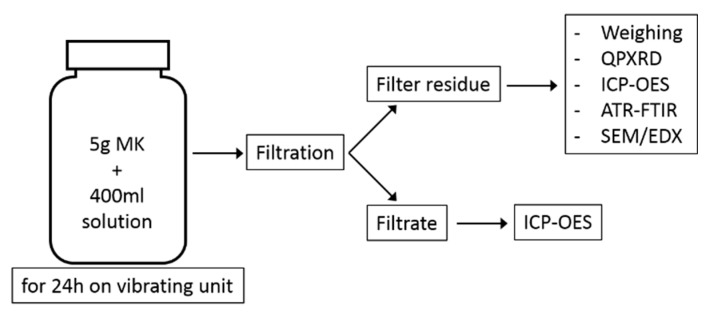
Schematic illustration of the test program.

The filtrate was acidified with concentrated HCl to a pH of 1 and filled up to 500 mL in a volumetric flask with distilled water, ready for ICP-OES analysis. A schematic summary of the experimental program is shown in Figure 2.

### 2.2. QPXRD

XRD measurements were performed with a PANalytical Empyrean diffractometer equipped with a primary Bragg-Brentano^HD^ monochromator and a PIXcel^1D^ linear detector (Malvern Panalytical, Malvern, UK). A diffractogram was taken from 4° to 42° 2Θ in continuous scan mode with a step size of 0.013 and a counting time of 25.5 s per step at 40 kV and 40 mA with CuKα radiation. Measurements were analyzed with the software HighScore 4.7. The sample was prepared with a back-loading tool and covered with a Kapton film (DuPont, Wilmington, DE, USA). The use of the Kapton film allows to summarize the instrumental background and the background caused by the Kapton film and ensures a stable refinement [38]. The quantifications were performed combining external standard [47,48] and PONKCS method [35,49]. Table 2 shows the structures used for Rietveld refinement of the crystalline components of the sample.

The calibrations for quantitative analysis of the hkl-phases were performed with ZnO as internal standard. The procedure for creating the hkl-phases, their calibration, as well as the refinement parameters, are described in detail in [38].

### 2.3. SEM/EDX

For the SEM/EDX analyses, the dried samples were embedded in epoxy resin, ground and polished with isopropanol after hardening, and finally coated with carbon. Since the habitus of the particles is not easily recognizable on embedded samples, a second set of samples was prepared by scattering the powder on two-component adhesives, followed by coating with gold.

The analyses were performed on a Zeiss Evo LS 15 (Zeiss, Oberkochen, Germany) equipped with an Oxford X-Max^N^ 50 EDX detector (Oxford Instruments, High Wycombe, UK) at 20 kV and a working distance of 8.5 mm. The EDX analyses were measured against a kaolinite standard (Processing Plant, Bugle, Cornwall, UK from Micro-Analysis Consultants Ltd.) of known composition. Each EDX spectrum was collected for 100 live seconds of accumulated count duration. In the EDX analyses, special attention was paid to the metakaolin particles and areas were selected for analysis in which no crystalline phases could be detected on the surface. Thus, the EDX analyses represent the chemical composition of the X-ray amorphous part of the sample (MK_Am_).

### 2.4. ICP-OES

The filter residue had to be solved for ICP-OES analysis in nitric acid after melt fusion using lithium metaborate. For this purpose, 0.4 g of the sample was weighed with 1.6 g lithium metaborate and melted at 950 °C in a platinum crucible in a muffle furnace. In the next step, the melted sample was quenched in 200 mL concentrated nitric acid (65 wt.% HNO_3_) and dissolved in an ultrasonic bath. Finally, the solution was filled up to 500 mL in a volumetric flask with distilled water.

The ICP-OES measurements were performed with a Varian 720 ES spectrometer (Agilent Technologies, Santa Clara, CA, USA) and evaluated with the software 1.1 supplied with the instrument. The measuring range for the respective element was adapted using a multi-point calibration with an external standard. The measurements and their evaluation were conducted according to [53]. The chemical composition of the X-ray amorphous content (MK_Am_) was calculated from the total analysis of the MK. The fractions of quartz (SiO_2_) and anatase (TiO_2_) determined by QPXRD were subtracted from the oxidic composition. For phengite, the individual oxidic components were calculated using an idealized phengite formula (KAl_1.5_Fe_0.5_(Al_0.5_Si_3.5_O_10_)(OH)_2_) and subtracted from the total analysis. Finally, the calculated oxides were normalized to 100%. This method provides comparable results to the EDX analysis and was used to cross check the EDX measurements. The deviation of the ICP results from the EDX analyses is ±1 wt.% for the main elements (>10 wt.%) and ±0.5 wt.% for the secondary elements (<10 wt.%).

### 2.5. ATR-FTIR

The ATR-FTIR analyses were performed on a ThermoFisher Scientific Nicolet iS10 FTIR spectrometer equipped with an EverGlo TM MIR radiation source (λ = 15,798 cm^−1^) and dTGS detector (Waltham, MA, USA). The spectra were measured in the wavenumber range from 400–4000 cm^−1^ with diamond as ATR crystal, collecting a series of 16 scans at a resolution of 4 cm^−1^. The evaluation of the data was carried out with Omnic 9.3 (ThermoFisher Scientific, Waltham, MA, USA).

### 2.6. Calculation of the Degree of Reaction

The degree of reaction was calculated with different approaches using the results from weighing the filter residue,

(1) $$R_{\mathrm{weight}}\left[\%\right]=\left[1-\frac{m_{\mathrm{filter}}-{\;m}_{\mathrm{cry}}}{m_{\mathrm{MKAm}}}\right]\times 100$$

the XRD quantification of the quartz,

(2) $$R_{\mathrm{Quartz}}\left[\%\right]=\left[1-\frac{m_{\mathrm{Quartz}}\times \frac{100}{c_{\mathrm{Quartz}}}-{\;m}_{\mathrm{cry}}}{m_{\mathrm{MKAm}}}\right]\times 100$$

the amorphous content (MK_Am_) and

(3) $$R_{\mathrm{MKAm}}\left[\%\right]=\left[1-\frac{m_{\mathrm{cry}}\times \frac{100}{\left(100-c_{\mathrm{MKAm}}\right)}-{\;m}_{\mathrm{cry}}}{m_{\mathrm{MKAm}}}\right]\times 100$$

from Si- and Al-solubility (R_Si/Al_ [%]).

The calculation of the degree of reaction from weighing the filter residue (m_filter_ [g]) (Equation (1)) is based on the assumption that the absolute amount of crystalline phases of the sample remains constant and does not dissolve during shaking. The mineralogical composition was used to calculate the absolute mass of the crystalline phases (m_cry_ = 0.35 g) and the weight of the MK_Am_ (m_MKAm_ = 4.65 g) in 5 g of the sample.

The calculations from the QPXRD results are also based on the assumption that the crystalline phases do not dissolve during the experiment. For the calculation of the reaction degree from the quartz content (Equation (2)), the absolute mass of quartz (m_Quartz_ = 0.25 g) in 5 g sample was used. The degree of reaction can be calculated directly from its quantification (c_Quartz_). For the calculation of the degree of reaction from the quantification of MK_Am_ (c_MKAm_) (Equation (3)), the total crystalline content of the sample (100 - c_MKAm_) was taken into account.

The degree of reaction from the ion solubility (R_Si/Al_) is calculated from the quotient of the sum of the dissolved Si- and Al-ions and the initial content of Si and Al in MK_Am_. The chemical composition of the ICP-OES analysis was used to calculate the initial content of Si and Al in MK_AM_ (2.3 g).

## 3. Results

### 3.1. Time Dependent pH Values of the Suspensions

Table 3 shows the time-dependent pH-values of the suspension. There are almost no measurable changes during the observation period. Previous investigations show that the measurement of pH values at very high concentrations with a glass electrode is faulty and is underdetermined due to the alkali error [54]. This effect occurs mainly with NaOH. Due to the logarithmic nature of the pH scale, even significant changes in concentration at very high concentrations cause only a slight change in the pH value. Accordingly, no conclusions can be drawn at this point from the results of the pH measurements at such high concentrations of KOH and NaOH.

### 3.2. Weighing the Filter Residue

Table 4 lists the masses of the filter residues determined after 24 h of dissolution, filtration, washing and drying. The given error was determined from the deviation of the mean value of the two individual determinations. As expected, the difference to the initial weight (5 g) is small for MK stored in distilled water. In contrast, the weight loss with the various alkaline solutions is very clear, with MK-NaOH showing by far the highest one, with approximately 3 g. A conventional presentation of the results in wt.% falsifies the results due to the presence of crystalline phases in the sample, which might not dissolve.

### 3.3. QPXRD

**Figure 3:**
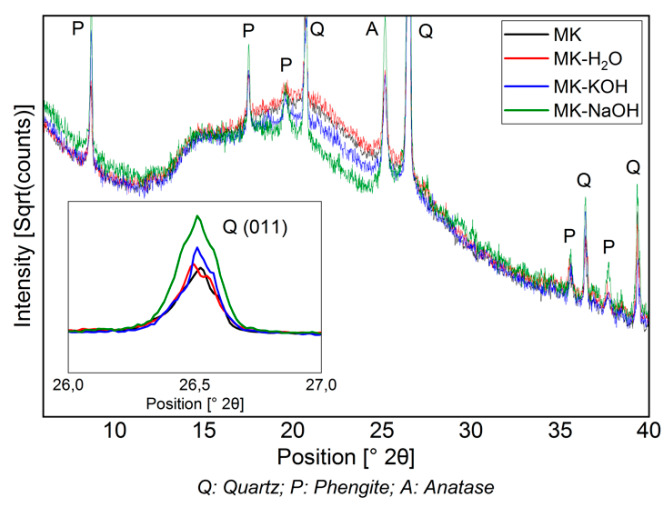
Diffractogram (CuKα) of MK compared to those of insoluble residues of MK treated with different solutions. The decrease in the X-ray amorphous hump and the enrichment of crystalline phases (quartz peak (011) is highlighted in the enlarged range between 26–27° 2Θ) can be seen.

Figure 3 shows the diffractograms of the filter residues. For the sake of a clearer overview, the MK-MOH measurement is not given. The intensity is expressed as a square root of the counts and the peaks of the crystalline phases are partially cut off. This emphasizes the changes in the X-ray amorphous hump. The diffractograms MK and MK-H_2_O are almost congruent. Thus, no structural change can be detected when the sample (MK) is treated with distilled water (MK-H_2_O). With MK-KOH, a slight decrease in the X-ray amorphous hump and a slightly higher maximum intensity of the peaks in the crystalline phases can be observed. This trend becomes more pronounced with MK-NaOH and leads to clearly discernible differences between MK and MK-NaOH. The increase in the intensity of the peaks of the crystalline phases is clearly visible at the (011)-reflex of the quartz, which is highlighted in the enlarged insert displaying a range from 26–27° 2Θ (Figure 3).

Table 5 summarizes the results of the XRD quantifications. An enrichment of all crystalline phases and a decrease in MK_Am_ from MK-MOH via MK-KOH to MK-NaOH can be observed.

### 3.4. Chemical Analysis

Table 6 shows the ion content of main elements in the filtrates obtained after the reaction of different solutions with MK. Distilled water treatment (MK-H_2_O) results in a low ion content for Si and Al. Here, the values for Fe and Ti are below the detection limit. The dissolved ion content increases with increasing alkali metal ion content of the alkaline solutions. It is noticeable that the content of Fe and Ti remains very low and changes only slightly with increasing pH, while Si- and Al-ion contents rise drastically.

Table 7 gives the results of the ICP-OES analyses of the filter residues. Since the values from the EDX analyses (Section 2.3) show only a small deviation from the ICP-OES results after correction of the crystalline phase content (Section 2.4), they are not shown additionally in the main text. The EDX results are available as supplementary Table S1. Overall, it can be stated that both the SiO_2_ and the Al_2_O_3_ content of MK_Am_ and MK_Am-H2O_ decreases via MK_Am-MOH_, MK_Am-KOH_ and MK_Am-NaOH_. The values for MK_Am-MOH_ and MK_Am-KOH_ are in a similar range, while MK_Am-NaOH_ shows a further and more pronounced decrease. This correlates with the increasing Si- and Al-ion solubility (Table 6). For Fe_2_O_3_ and TiO_2_, where hardly any ion solubility can be measured (Table 6), the trend is opposite and an enrichment takes place. Furthermore, an uptake of alkalis can be detected, corresponding to the alkalinity of the solution used.

### 3.5. ATR-FTIR

**Figure 4:**
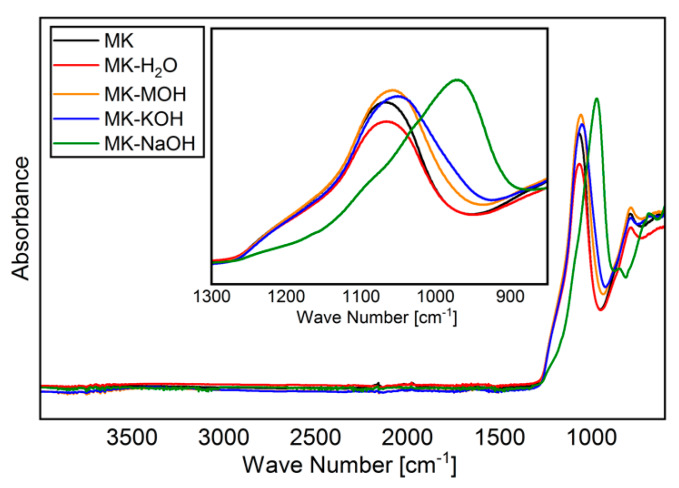
FTIR spectra of all samples analyzed.

The FTIR spectra of MK and residues analyzed are shown in Figure 4. As reported elsewhere [45,55,56], a broadened band is visible in the region of Si-O vibration (900–1200 cm^−1^), owing to amorphization of the crystalline kaolinite structure. Special attention will be paid to this band (zoomed region in Figure 4 from 850 to 1300 cm^−1^). As expected, MK and MK-H_2_O behave similarly, and no differences can be found within the range of reproducibility of the measurements. With MK-MOH and MK-KOH, a slight shift in the band to smaller wave numbers and a broadening of the peak can be observed. MK-NaOH leads to a clear shift in the band to smaller wave numbers. Table 8 summarizes the determined wave numbers and the corresponding full width at half maximum (FWHM).

### 3.6. SEM Images

**Figure 5:**
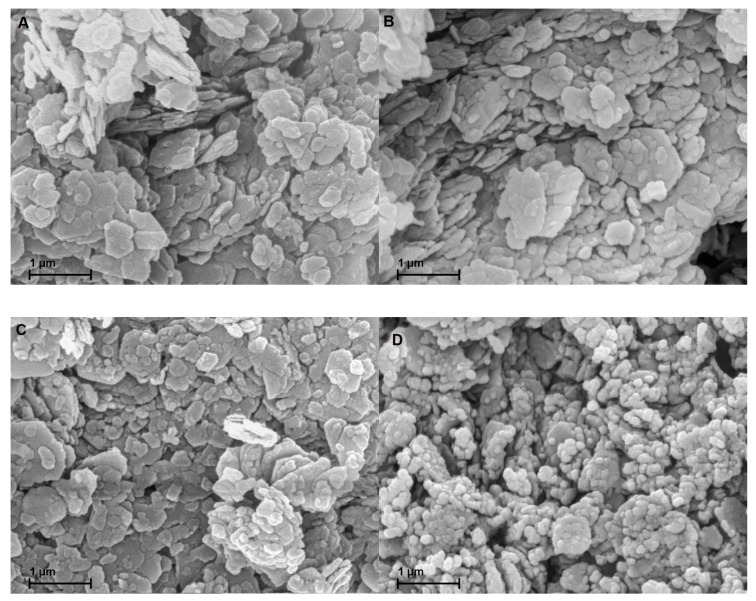
SEM images of the samples coated with gold of (**A**) MK; (**B**) MK-H_2_O; (**C**) MK-KOH; (**D**) MK-NaOH.

Figure 5 A-D display the SEM images of the gold coated samples. Owing to the similarity of MK-MOH and MK-KOH, only MK-KOH is shown. There are no optical differences visible between MK and MK-H_2_O. The treatment in distilled water has no effect on the shape, size and habitus of the metakaolin particles. Differences are also very small in comparison to MK-KOH. On closer inspection, a reduction in particle size caused by the dissolution process can be implied. Clear differences become obvious for MK-NaOH. Here, an alteration of the metakaolin particles takes place. The dissolution process has progressed so far that the mean particle size decreases and the morphology of many particles has changed significantly.

### 3.7. Calculations of the Degree of Reaction

**Figure 6:**
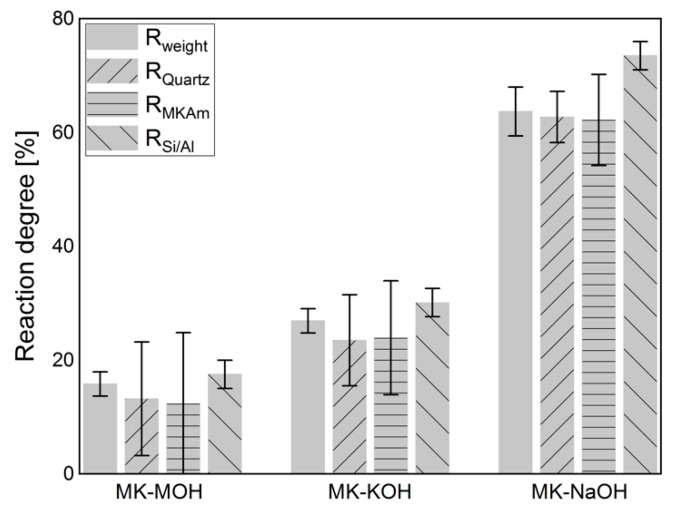
Degree of reaction calculated according to different approaches (Section 2.6).

Figure 6 displays the results of the calculations of the degree of reaction based on the weighing of the filter residue (Table 4), the QPXRD (Table 5) and the Si/Al-solubility (Table 6). The calculation of the degree of reaction of R_weight_ (Equation (1), Section 2.6) considers the crystalline phases in comparison to the weighing of the filter residue (Table 4). The differences in mass are related exclusively to the X-ray amorphous fraction and thus reflect the mass loss in percent. Since errors in the quantification of one phase do not affect the quantification of another phase when calculating the phase contents following the G-factor method [48,57], the degree of reaction was determined from both the quantification of the quartz and of the MK_Am_. An increase in the degree of reaction from MK-MOH via MK-KOH to MK-NaOH can be observed. In all systems, the degree of reaction calculated from the Si/Al-solubility is the highest, while the degrees of reaction calculated from XRD quantifications are the lowest. For the calculation of the degree of reaction from the results of the XRD quantifications (R_Quartz_ and R_MKAM_) a large error in the degrees of reaction occurs. This effect is particularly pronounced with MK-MOH and MK-KOH due to higher error propagation at lower reaction rates. Thus, for MK-NaOH the error in the degree of reaction is significantly lower. This is in line with observations in the literature, which also find a large error in the degree of reaction calculated from XRD quantifications for low reaction rates or low SCM contents [26,36]. Another reason for the large errors in the degree of reaction calculated from the XRD quantifications results from the experimental setup, as the degree of reaction does not reflect the decrease in MK_AM_ in the sample. A degree of reaction of 12.3% (MK-MOH) only leads to a difference in MK_AM_ of 0.9 wt.% and thus to large errors in the calculated degree of reaction based on XRD quantifications of MK_AM_ with an accuracy of ±1 wt.%. Due to the strong error propagation from the XRD quantifications, the data obtained must be interpreted with caution. This is also known from the calculation of the degree of reaction with results of other test methods which exhibit small deviations of the determined contents. Scrivener et al. [58] report, for instance, a possible relative error of ±50%, when calculating the degree of reaction from the CH consumption determined by TG. The absolute differences in the degree of reaction (ΔR) increase with increasing degree of reaction, while the relative differences (ΔR/R_max_) decrease (Table 9).

## 4. Discussion

### 4.1. Changes of MK_Am_

**Figure 7:**
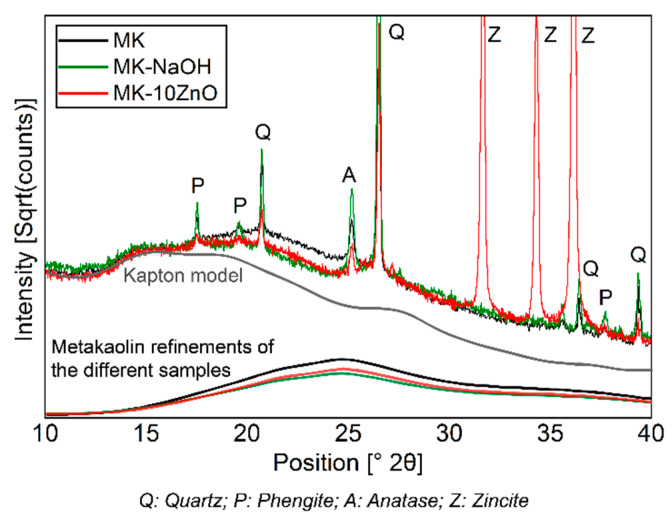
Comparison of diffractograms of MK-NaOH and a MK sample mixed with 10 wt.% ZnO as internal standard. The scattering contribution to the diffractogram of MK_Am_ of the different samples and of the Kapton model is shown.

The dissolution of MK_Am_ leads to a decrease in the X-ray amorphous hump (Figure 3) and thus a corresponding enrichment of the crystalline phases since these phases are not dissolved. A comparison of the scattering contribution of the X-ray amorphous hump between MK-NaOH and a MK sample mixed with 10 wt.% ZnO as internal standard (MK-10ZnO; MK_Am_ = 83.7 wt.%) confirms this observation. There is only a small difference in the diffractogram between MK-10ZnO and MK-NaOH in the area from 15°–30° 2Θ, where the scattering contribution of metakaolin is visible (Figure 7). Thus, the quantification of MK-NaOH is reliable since the result (83.4 wt.%) corresponds with the MK_Am_ content of MK-10ZnO. Other effects, like, for instance, geopolymerization, can be ruled out. According to Williams [59] geopolymerization would result in a clear shift in the X-ray amorphous hump. No such effect and thus no geopolymer formation can be detected from the XRD data. Additional thermogravimetric analyses show only a small mass loss (<1 wt.%) for all samples. Thus, the high water to solid ratio of 80 is sufficient to avoid condensation of geopolymers. The studies of Kaps et al. [11,60] confirm this. According to Palomo, et al. [61], geopolymers are formed in several stages. The contact of aluminosilicates with high pH solutions leads to the dissolution of Si- and Al- monomers, which in turn interact and form dimers, trimers and so on. If a saturation point is exceeded, geopolymers condense. This saturation point is not reached with the selected high water to solids ratio [33].

Based on the results of R_Si/Al_ of MK-NaOH given in Figure 6, the MK_Am_ content in the residue should be 78 wt.%. Here, the uptake of alkalis seems to have a direct influence on the diffractogram, namely increasing the scattering contribution of metakaolin compared to the dissolved Si- and Al- monomers. Pore solution tests on cement paste [62,63,64,65,66] in cementitious systems yield, in comparison, a significantly reduced availability of alkalis (c(Na) ≈ 50 mmol/L, c(K) ≈ 450 mmol/L [64]), and thus the effect should be reduced on the diffractogram which is caused by alkali uptake. As a result, the quantification of metakaolin in cementitious systems is influenced only to a minor extent by alkali uptake. Quantifications of the degree of reaction of metakaolin in cementitious systems with the PONKCS method [28] also confirm this assumption. An accurate modelling and calibration of the X-ray amorphous content as well as a precise description of the background [38] seems to be more important for a reliable quantification with the PONKCS method.

**Figure 8:**
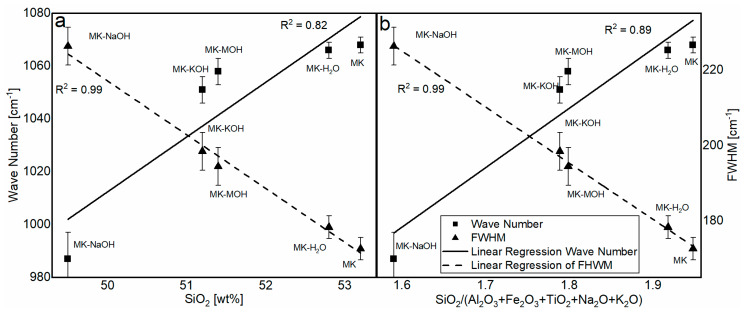
Correlation of the SiO_2_ content (**a**) and the molar ratio of SiO_2_/(Al_2_O_3_ + Fe_2_O_3_ + TiO_2_ + Na_2_O + K_2_O) (**b**) in the aluminosilicate structure with the wavenumber and FWHM.

The analysis of the FTIR data reveals a significant structural change in the MK_Am_. A shift in the position of the Si-O band maximum as well as a broadening of the peak can be observed. The related literature [45,67] reports a shift in this band to smaller wave numbers depending on the silicon content in the aluminosilicate structure. The dependence of the wave number (Table 8) on the silicon content (Table 7) is elaborated in Figure 8a. There is a good correlation between the silicon content and the shift in the band. A similar correlation is given for the molar ration of SiO_2_/(Al_2_O_3_ + Fe_2_O_3_ + TiO_2_ + Na_2_O + K_2_O) (Figure 8b). The enrichment of Fe_2_O_3_ and TiO_2_, as well as the uptake of alkalis, might influence the chemical environment of the Si-O band. A possible increase in the bond length of the Si-O band might induce the shift to lower wavenumbers. The FWHM can indicate the degree of disorder within a structure. Disordered structures show a broader peak than ordered structures [45,68]. Since parts of the kaolinite structure remain intact during calcination and transformation into metakaolin [39], the broadening of the peak can be interpreted as additional defects in the X-ray amorphous structure after treatment in alkaline solution according to Król et al. [45]. This effect can also be observed here. It is assumed that the ionic radii in 6-fold coordination with oxygen of Na^+^ (116 pm) and K^+^ (152 pm), which are significantly larger than Si^4+^ (54 pm) and Al^3+^ (53 pm) [69], also affect and additionally disorder the structure of the metakaolin. This highlights the correlation of SiO_2_/(Al_2_O_3_ + Fe_2_O_3_ + TiO_2_ + Na_2_O + K_2_O) with the FWHM (Figure 8b). The SiO_2_ content shows a just as good correlation with the FWHM (Figure 8a). However, it is assumed that the broadening of the peak is less due to the SiO_2_ content than the enrichment of Fe_2_O_3_ and TiO_2_, as well as the uptake of alkalis. Garg and Skibsted [43] showed by NMR measurements before and after dissolution in alkaline solution that 5-fold coordinated Al dissolves preferably and conclude a higher structural stability for 4-fold coordinated Al. These modifications have an influence on the binding conditions of the metakaolin structure and thus could influence the position of the FTIR bands.

SEM investigations demonstrate the influence of the dissolution of the Si- and Al-monomers on the shape of the particles. A dissolution process of the particles seems to take place. The enrichment of iron and titanium allows the conclusion that areas enriched with iron and titanium (Table 7) are hardly or not at all dissolved. As a result, metakaolin particles are not evenly dissolved from their edges and the SEM image of MK-NaOH (Figure 5D) could suggest the disintegration of the particles. Further investigations of the metakaolin particles in a transmission electron microscope could provide information about the element distribution within the particles. Inhomogeneous distribution of cations in the metakaolin structure could support the mentioned hypothesis.

Overall, the contribution of metakaolin to the pozzolanic reaction seems to be a congruent dissolution process. The almost constant SiO_2_/Al_2_O_3_ molar-ratio of about 2 (Table 7) in all investigated samples confirms that all areas are dissolved congruently and that neither Si- nor Al- ions are preferred. Thus, the structural changes seem to only slightly modify the scattering contribution of metakaolin to the diffractogram and the dissolution of metakaolin in alkaline solution, respectively, in cementitious systems is reflected in the decreasing X-ray amorphous hump in the diffractogram. In case of congruent dissolution, the same hkl-phase model of metakaolin can be used for quantification before and after treatment in alkaline solutions and thus enables a reliable quantification during cement hydration. Snellings [70] describes a shift in the X-ray amorphous hump towards lower angles 2Θ depending on the SiO_2_ content of synthesized calcium aluminosilicate glasses. In case of an incongruent dissolution process of metakaolin calcined at higher temperatures (>900 °C) [43], an enrichment of or reduction in the SiO_2_ content could also cause a shift in the X-ray amorphous hump. Such behavior could not be quantified with one hkl-phase model for metakaolin and would require the use of different hkl-phase models. As already described in the literature [26,28,36,37,58], the PONKCS method offers a powerful opportunity to investigate the influence of X-ray amorphous SCM on the hydration of cements.

### 4.2. Differences of the Degree of Reactions

**Figure 9:**
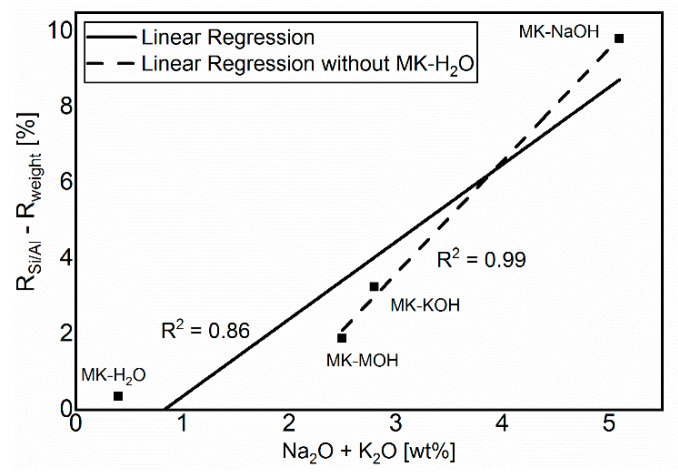
Correlation between the sum of alkalis and the difference of the calculated degrees of reaction.

The comparison in Figure 6 of R_weight_ and R_Si/Al_ shows a lower degree of reaction for R_weight_. The difference is due to the uptake of alkalis in MK_Am_. The additional alkalis lead to a higher weight of the filter residue than calculated from the Si/Al-solubility only, and thus to a lower degree of reaction based on R_weight_ in comparison to R_Si/Al_. Consequently, the deviations between the two degrees of reaction increases with increasing alkali uptake. This relationship is illustrated in Figure 9. MK-H_2_O is somewhat out of the range, because, on the one hand, the degree of reactions and thus the measurable effects are very low and, on the other hand, no alkalis are available for uptake in the distilled water. Without MK-H_2_O, the correlation fits very well and confirms the aforementioned.

When comparing the degrees of reaction within an alkaline solution, it is noticeable that R_Si/Al_ is the highest for all investigated systems. From the evaluation of the weight of the filter residue and the QPXRD data, their lower calculated degree of reaction seems to be connected. Both the additional weight and the increased scattering contribution in the diffractogram appear to be related to the uptake of alkalis. This relationship is particularly evident for MK-NaOH. There is only a very small deviation in the calculated degrees of reaction between R_weight_ and R_Quartz_ or R_MKAm_ (Figure 6).

More obvious differences exist between R_weight_ and R_MKAm_ or R_Quartz_ for lower reaction degrees such as for MK-MOH and MK-KOH. A reason for this is the small quantitative difference in the MK_Am_ content between the initial sample and the samples MK-MOH and MK-KOH after the test. Even small deviations in the quantification of the MK_Am_ or quartz content (±1 or ±0.5 wt.%, Table 5) lead to significant errors in the calculation of the degree of reaction due to the experimental setup and error propagation (Figure 6). Avet, Li and Scrivener [26] and Snellings, Salze and Scrivener [36] showed in their investigations in cementitious systems that errors in the determination of the reacted metakaolin with the PONKCS method could lead to pronounced errors in the determination of the degree of reaction. This effect was also reported for other SCM like fly ash and slag [23]. Due to the resulting large errors, the data must be interpreted with care. Overall, the resulting trends appear to be consistent. The present investigations are not dealing with a hydrating system, but the XRD quantifications are performed before and after a dissolution process of metakaolin in alkaline solution. In contrast to Avet et al. [26], the results of the degree of reaction cannot be determined directly from the decrease of MK_AM_. Thus, the results represent a comparison between the degree of reaction from the Si- and Al-solubility and the PONKCS quantifications, but the procedure cannot be transferred to hydrating systems. Nevertheless, these comparisons are important to evaluate and confirm the reliability of the PONKCS quantifications in reacting systems, as the reaction of metakaolin in hydrating systems is also a dissolution process.

The degrees of reaction in highly alkaline solutions determined here do not allow straightforward conclusions to be drawn about the degree of reaction of the SCM in cementitious systems. But the significantly higher solubility of Si and Al for MK-NaOH in contrast to MK-KOH leads to the assumption that a higher degree of reaction of metakaolin may be expected in cements with a high sodium content in the pore solution. However, a classification of the reactivity of clays due to Si and Al solubility in NaOH solution with different metakaolin content [12] as well as of calcined clays and different phyllosilicates, seems possible and plausible [43,44,71]. This is in line with Maier, et al. [72], who found that Si- and Al-solubility, such as heat of hydration determined by R^3^ reactivity test [17], exhibit both comparable correlation with the total Al_2_O_3_ content as well as the kaolinite content of the clays.

## 5. Conclusions

The correlation of dissolved silicon and aluminum from dissolution experiments with XRD quantification on MK after treatment in alkaline solutions offers new insights into the possibilities and limitations of quantifying MK during pozzolanic reaction with the PONKCS method. After systematic investigations of the filtrate and the filter residue, the following conclusions can be drawn.

A change in the binding condition of metakaolin owing to the treatment in alkali solutions can be proven using ATR-FTIR. The enrichment of iron and titanium as well as the uptake of alkalis in the metakaolin structure seem to cause a shift in the Si-O band to smaller wave numbers and a broadening of the bands.

The participation of the metakaolin in the pozzolanic reaction is a congruent dissolution process of the Si- and Al-monomers to a large extent. There seems to be an inhomogeneous distribution of the cations in the particles. Iron- and titanium-rich areas in the particles do not seem to dissolve and thus cause the particles to disintegrate.

These observations affect the intensity of the scattering contribution of the MK_Am_ in the diffractogram. However, these structural changes do not cause any change in the position of the X-ray amorphous hump if the dissolution of the metakaolin particles is congruent. Therefore, it is reasonable to use one hkl-phase model for the quantification of metakaolin calcined at its optimum temperature between 500 and 800 °C before and after dissolution in alkaline solution as well as during the hydration of cementitious systems.

The effect of the uptake of alkalis on the intensity of the X-ray amorphous hump results in a difference in XRD quantifications compared to the expected value calculated from the dissolved Si- and Al-ions. This effect can be measured for highly alkaline solutions on a pure metakaolin sample and leads to differences in the calculation of the degree of reaction. For common substitution rates of metakaolin in cementitious systems, where the content of alkalis in the pore solution is rather low, the effect on the quantification of MK_Am_ with the PONKCS method seems to be negligible or at least below the error of the quantification. Accurate modelling and calibration, as well as a very precise description of the background, is more decisive for the success of the quantification. Consequently, the PONKCS method is a suitable method for investigating the quantity and reaction mechanisms of the X-ray amorphous SCMs involved in the hydration of cements. The strong effect of error propagation for low degrees of reaction and low substitution rates in cementitious systems remains a problem when calculating the degree of reaction based on quantifications using the PONKCS method.

## Figures and Tables

**Table 1:** Chemical composition, loss on ignition (LOI) and mineralogical composition of metakaolin (MK) [38,44].

Oxides (wt.%)	MK	Phases (wt.%)	MK
SiO_2_	54.5	Quartz	5.0
Al_2_O_3_	40.2	Anatase	0.6
Fe_2_O_3_	1.8	Phengite	1.4
CaO	<0.1	X-ray amorphous	93.0
MgO	0.2		
SO_3_	<0.1		
Na_2_O	0.3		
K_2_O	0.3		
TiO_2_	1.4		
LOI	1.3		

**Table 2:** Inorganic Crystal Structure Database (ICSD) structure data for the Rietveld refinement.

Phase	Author	ICSD-No.
Quartz	Le Page and Donnay [50]	174
Anatase	Horn, et al. [51]	9854
Phengite	Ivaldi, et al. [52]	158072

**Table 3:** Time dependent pH-values of the different MK suspensions.

	MK-H_2_O	MK-MOH	MK-KOH	MK-NaOH
pH after 5 min	5.8	13.5	14.1	13.1
pH after 30 min	6.0	13.5	14.0	13.0
pH after 6 h	6.0	13.4	14.0	12.9
pH after 24 h	6.2	13.5	14.1	13.1

**Table 4:** Results of weighing the filter residue after dissolution of 5 g MK in different solutions in g.

MK-H_2_O	MK-MOH	MK-KOH	MK-NaOH
4.93 ± 0.05	4.27 ± 0.10	3.75 ± 0.10	2.04 ± 0.20

**Table 5:** Results of QPXRD in wt%. The absolute error is ±0.5 wt.% for the crystalline phases and ±1 wt.% for MK_AM_.

Phase	MK	MK-H_2_O	MK-MOH	MK-KOH	MK-NaOH
Quartz	5.0	5.2	5.6	6.3	12.0
Anatase	0.6	0.6	0.6	0.8	1.1
Phengite	1.4	1.5	1.7	2.1	3.1
MK_Am_	93.0	92.8	92.1	90.8	83.4

**Table 6:** Ion content [mg] of the filtrate measured with ICP-OES (after the experiment of 5 g sample in 400 mL solution).

Elements	MK-H_2_O	MK-MOH	MK-KOH	MK-NaOH
Si	24	211	353	866
Al	19	191	341	828
Fe	- *	7	9	15
Ti	- *	3	5	8

* Detection limit is 0.5 mg.

**Table 7:** Chemical composition [wt.%] of MK_Am_ left after dissolution with different solvents of all samples measured with ICP-OES and the molar ratio of SiO_2_/Al_2_O_3_ and SiO_2_/(Al_2_O_3_ + Fe_2_O_3_ + TiO_2_ + Na_2_O + K_2_O).

Oxides	MK_Am_	MK_Am-H2O_	MK_Am-MOH_	MK_Am-KOH_	MK_Am-NaOH_
SiO_2_	53.2	52.8	51.4	51.2	49.5
Al_2_O_3_	43.7	43.6	42.3	42.2	40.0
CaO	0.1	0.1	0.1	0.1	0.1
Fe_2_O_3_	1.5	1.8	2.1	2.1	3.1
K_2_O	0.0	0.0	2.0	2.7	0.0
MgO	0.2	0.3	0.2	0.1	0.2
Na_2_O	0.3	0.4	0.5	0.1	5.1
TiO_2_	0.9	0.9	1.5	1.4	2.0
SiO_2_/Al_2_O_3_	2.07	2.05	2.06	2.06	2.10
*	1.95	1.92	1.80	1.79	1.59

* SiO_2_/(Al_2_O_3_ + Fe_2_O_3_ + TiO_2_ + Na_2_O + K_2_O)

**Table 8:** Summary of the determined wavenumbers and FWHM of the broadened Si-O band.

Sample	Wavenumber [cm^−1^]	FWHM [cm^−1^]
MK	1068 ± 3	173 ± 3
MK-H_2_O	1066 ± 3	178 ± 3
MK-MOH	1058 ± 3	194 ± 5
MK-KOH	1051 ± 3	198 ± 5
MK-NaOH	982 ± 10	226 ± 5

**Table 9:** Summary of the minimal (R_min_) and maximal (R_max_) degree of reaction, their absolute difference (ΔR) and their relative difference (ΔR/R_max_).

	R_min_	R_max_	Absolute Difference ΔR [%]	Relative Difference ΔR/R_max_ [%]
MK-MOH	12.3	17.5	5.2	30.0
MK-KOH	23.5	30.1	6.6	22.0
MK-NaOH	62.2	73.5	11.3	15.4

## Supplementary Materials

The following are available online at https://www.mdpi.com/1996-1944/13/10/2214/s1, Table S1: Chemical composition [wt.%] of MK_Am_ left after dissolution with different solvents of all samples measured with EDX and the molar ratio of SiO_2_/Al_2_O_3_ and SiO_2_/(Al_2_O_3_ + Fe_2_O_3_ + TiO_2_ + Na_2_O + K_2_O).
