# Supplementary material for: Reactivity of Metakaolin in Alkaline Environment: Correlation of Results from Dissolution Experiments with XRD Quantifications
Source: Materials (Basel). 2020 May 12;13(10):2214. doi: 10.3390/ma13102214 (PMC7287791; doi:10.3390/ma13102214)
Supplement: Supplementary file 1 [file materials-13-02214-s001.pdf]

materials

MDPI

Supplementary

Reactivity of Metakaolin in Alkaline Environment: Correlation of Results from Dissolution Experiments with XRD Quantifications

Sebastian Scherb [1,*], Mathias Köberl [1], Nancy Beuntner [1], Karl-Christian Thienel [1] and Jürgen Neubauer [2]

1 Civil Engineering and Environmental Science, Universitätder Bundeswehr Munich, Werner-Heisenberg-Weg 39, Neubiberg 85579, Germany; mathias.koeberl@unibw.de (M.K.); nancy.beuntner@unibw.de (N.B.); christian.thienel@unibw.de (K.-C.T.)

2 GeoZentrum Nordbayern,Mineralogy, Friedrich-Alexander Universitaet Erlangen-Nuernberg, Schlossgarten 5a, Erlangen 91054, Germany; juergen.neubauer@fau.de

* Correspondence: sebastian.scherb@unibw.de

Received: 15 April 2020; Accepted: 11 May 2020; Published: 12 May 2020

Table S1. Chemical composition [wt.%] of MK_{Am} left after dissolution with different solvents of all samples measured with EDX and the molar ratio of SiO_2/Al_2O_3 and $SiO_2/(Al_2O_3 + Fe_2O_3 + TiO_2 + Na_2O + K_2O)$.

Oxides	MK_{Am}	$MK_{Am\text{-}H2O}$	$MK_{Am\text{-}MOH}$	$MK_{Am\text{-}KOH}$	$MK_{Am\text{-}NaOH}$
SiO_2	53.9	53.6	52.0	51.7	48.7
Al_2O_3	43.2	43.4	41.8	42.0	39.4
CaO	0.1	0.1	0.1	0.1	0.1
Fe_2O_3	1.6	1.4	2.3	1.8	3.6
K_2O	0.0	0.0	2.0	3.1	0.0
MgO	0.1	0.2	0.1	0.1	0.2
Na_2O	0.3	0.3	0.4	0.1	5.5
TiO_2	0.7	1.0	1.3	1.1	2.4
SiO_2/Al_2O_3	2.12	2.10	2.09	2.10	2.11
*	2.00	1.98	1.85	1.82	1.54

* $SiO_2/(Al_2O_3 + Fe_2O_3 + TiO_2 + Na_2O + K_2O)$.
